# Evaluation of computational phage detection tools for metagenomic datasets

**DOI:** 10.3389/fmicb.2023.1078760

**Published:** 2023-01-25

**Authors:** Kenneth E. Schackart, Jessica B. Graham, Alise J. Ponsero, Bonnie L. Hurwitz

**Affiliations:** ^1^Department of Biosystems Engineering, The University of Arizona, Tucson, AZ, United States; ^2^BIO5 Institute, The University of Arizona, Tucson, AZ, United States; ^3^Human Microbiome Research Program, Faculty of Medicine, University of Helsinki, Helsinki, Finland

**Keywords:** microbiome, bacteriophage, computational biology, benchmark, metagenome, virome

## Abstract

**Introduction:**

As new computational tools for detecting phage in metagenomes are being rapidly developed, a critical need has emerged to develop systematic benchmarks.

**Methods:**

In this study, we surveyed 19 metagenomic phage detection tools, 9 of which could be installed and run at scale. Those 9 tools were assessed on several benchmark challenges. Fragmented reference genomes are used to assess the effects of fragment length, low viral content, phage taxonomy, robustness to eukaryotic contamination, and computational resource usage. Simulated metagenomes are used to assess the effects of sequencing and assembly quality on the tool performances. Finally, real human gut metagenomes and viromes are used to assess the differences and similarities in the phage communities predicted by the tools.

**Results:**

We find that the various tools yield strikingly different results. Generally, tools that use a homology approach (VirSorter, MARVEL, viralVerify, VIBRANT, and VirSorter2) demonstrate low false positive rates and robustness to eukaryotic contamination. Conversely, tools that use a sequence composition approach (VirFinder, DeepVirFinder, Seeker), and MetaPhinder, have higher sensitivity, including to phages with less representation in reference databases. These differences led to widely differing predicted phage communities in human gut metagenomes, with nearly 80% of contigs being marked as phage by at least one tool and a maximum overlap of 38.8% between any two tools. While the results were more consistent among the tools on viromes, the differences in results were still significant, with a maximum overlap of 60.65%. Discussion: Importantly, the benchmark datasets developed in this study are publicly available and reusable to enable the future comparability of new tools developed.

## 1. Introduction

Prokaryotic viruses called bacteriophages (phages) are the most abundant biological entity in most ecosystems (Ofir and Sorek, 2018) and profoundly impact the ecology of natural ecosystems (Breitbart and Rohwer, 2005; Blazanin and Turner, 2021). For example, marine viruses have massive effects on ocean biochemistry, influencing nutrient cycling and carbon sequestration by altering host-driven processes through controlling bacterial population growth and altering metabolic function (as reviewed in Fuhrman, 1999; Hurwitz and U’Ren, 2016; Breitbart et al., 2018). Additionally, recent studies have demonstrated the importance of phages in shaping the human microbiota and interacting with human health (as reviewed in Edlund et al., 2015; Manrique et al., 2017; Sharma et al., 2018; Li et al., 2021). Next-generation sequencing techniques enable the identification of an exponential number of novel phages but also allow for a better understanding of phage populations in multiple ecosystems (Breitbart et al., 2002).

Despite the increasing number of virome studies, identifying viral sequences in metagenomic datasets is still computationally challenging. Since viruses lack a universal gene marker (e.g., 16S rRNA in prokaryotes), earlier bioinformatics methods to identify viruses from metagenomes often relied on sequence alignment methods against reference genome databases. Strikingly, in gut viromes, 75–99% of viral reads do not produce significant alignments to any known viral genome. This large range in alignable sequences can be partially explained by the high diversity, fast phage evolution, and their ability to integrate into their host genome and be mistaken as bacterial. Indeed, an inherent limitation to genome comparison approaches is the database completeness and clear separation between host and viral DNA. Moreover, these reference-based methods typically cannot identify novel phage sequences. To address these limitations, several dedicated computational tools and approaches were proposed. In 2015, the tool VirSorter (Roux et al., 2015) was released, identifying phage sequences by enrichment of viral hallmark genes, depletion of cellular genes indicated by reduced Pfam hits, and strand shifts. In 2016, MetaPhinder took into account the mosaicism of phage sequences by integrating hits to multiple genomes to classify a sequence as host or viral (Jurtz et al., 2016).

Recently, bioinformatic tools leverage machine learning algorithms to identify features of viral origin, and typically allow for a broader recall of previously unknown sequences than alignment-based approaches. Chosen features are genes and gene density (Amgarten et al., 2018; Antipov et al., 2020; Kieft et al., 2020; Tisza et al., 2020; Guo et al., 2021) and protein families (Amgarten et al., 2018) that are used to train classification models including random forest (Amgarten et al., 2018; Guo et al., 2021), naive Bayes (Antipov et al., 2020), and neural network (Kieft et al., 2020). Interestingly, some authors also proposed using the differential *k*-mer (short sequences of length *k*) frequencies between phages and prokaryotes for sequence classification (Deaton et al., 2017; Ren et al., 2017). These methods allow the detection of shorter phage sequences, as they do not require multiple open reading frames (ORF) for classification that are difficult to obtain in fragmentary metagenomic data. However, the classification results and the rationale behind the classification are typically difficult to interpret.

All in all, between 2015 and 2021, we identified 19 published tools designed for detecting phage in metagenomes, making the development of benchmarking datasets critical for exploring the limitations and biases of the currently available tools but also facilitating future tool development. Similar benchmark efforts are currently available for other computational tasks such as metagenome assembly, binning, and taxonomic profiling (Sczyrba et al., 2017; Meyer et al., 2019). Recently, several efforts to benchmark phage detection tools have been published, and explore the ability of these tools to correctly identify and classify dsDNA viruses and curate auxiliary metabolic genes (Pratama et al., 2021; Ho et al., 2022). However, the potential impact of parameters such as the sequence length, sequencing error, eukaryotic contamination, quality of assembly, and phage taxonomy on the tool’s classification performance is not explored.

In this study, we developed a series of benchmark datasets, each aiming at assessing a precise classification challenge in detecting phage in metagenomic datasets and evaluated phage metagenomic detection tools published before July 2021. Notably, this work only evaluated self-contained computational tools and did not include more modular viral discovery pipelines such as the IMG/VR viral discovery pipelines (Paez-Espino et al., 2017). Additionally, this work does not include tools specifically intended to detect integrated prophage in complete bacterial genomes, for which prior benchmarking efforts are already available (Roach et al., 2022). Importantly, we ensured the availability and reusability of the developed benchmark datasets and described how researchers could utilize them for benchmarking new phage detection tools (doi.org/10.5281/zenodo.7194616).

## 2. Materials and methods

### 2.1. Phage detection tools

Tools were categorized into two broad groups. Homology-based tools are those that utilize a reference database at the time of classification to search for homologues. Sequence-based tools are those that classify using a model trained on sequence features such as *k*-mer frequencies.

Each of the evaluated tools included in this study was installed from the recommended source following the authors’ instructions. When tools were available from several sources, Bioconda was preferred due to simplified dependency management. Tools that could not be obtained through Bioconda were directly cloned from GitHub or Sourceforge. Table 1 summarizes the tool and version number when available, the category, classification method, training of reference database, and how tools were obtained. Tools with “-” under distribution were not used for further benchmarking, and reasons for doing so are presented in Results.

**TABLE 1:** Overview of published metagenomic phage detection tools.

Tool and version	Category	Method	Training set/Reference database	Distribution	References
DeepVirFinder (1.0)	Sequence	*k*-mer based deep learning neural net	NCBI RefSeq genomes before May 2015 and virome sequences	GitHub	Ren et al., 2020
MARVEL (0.2)	Homology	Random forest utilizing gene density, strand shifts, and proteins.	NCBI RefSeq genomes before 2016	GitHub	Amgarten et al., 2018
MetaPhinder	Homology	Integrated analysis of BLASTn hits to a phage database	Viral dataset from NCBI genomes, EMBL EBI genomes, phageDB, PhAnToMe/bacterial dataset from NCBI genomes. Downloaded before August 2014	GitHub	Jurtz et al., 2016
PhaMers	Sequence	*k*-Nearest neighbors and centroid proximity metric of *k* mers	NCBI RefSeq genomes before October 2015	−	Deaton et al., 2017
PPR-Meta	Sequence	Convolutional neural network (CNN) of one-hot encodings of nucleobases and codons	NCBI RefSeq genomes. Download date unknown.	−	Fang et al., 2019
RNN-VirSeeker	Sequence	Long short-term memory (LSTM) of sequences	NCBI RefSeq genomes downloaded before January 2014.	−	Liu F. et al., 2022
Seeker	Sequence	LSTM of sequences	NCBI genomes and EMBL EBI genomes. Download date unknown.	PyPi	Auslander et al., 2020
Unlimited Breadsticks	Homology	HMM of virus hallmark genes	−	GitHub	Tisza et al., 2020
VIBRANT (1.0.1)	Homology	Neural network of protein signatures including ratios of KEGG, VOG, and PFAM hits, and presence of key viral-like genes.	NCBI RefSeq and Genbank before July 2019	Bioconda	Kieft et al., 2020
viralVerify (1.1)	Homology	Naive Bayes classifier using an hmmsearch of genes predicted with Prodigal	NCBI RefSeq genomes. Download date unknown.	Bioconda	Antipov et al., 2020
ViraMiner	Sequence	CNN of one-hot encoded nucleobases	Sequences from 19 WGS metagenomes from human microbiome samples.	−	Tampuu et al., 2019
VirFinder (1.1)	Sequence	Logistic regression using *k*-mers	NCBI RefSeq genomes downloaded before January 2014.	Bioconda	Ren et al., 2017
VirMine	Homology	BLAST search of ORFs against viral and non-viral databases	Viral dataset: NCBI RefSeq viral genomes. Bacterial dataset: Bacterial COGs. Download date unknown.	−	Garretto et al., 2019
VirMiner	Homology	Random forest (RF) based on functional profiling and protein homology	Viral dataset NCBI genomes and ACLAME database. Bacterial dataset: NCBI genomes. Downloaded October 2016.	−	Zheng et al., 2019
VirNet	Sequence	Deep attention model of sequences	NCBI RefSeq genomes. Download date unknown.	−	Abdelkareem et al., 2018
VIROME	Homology	Functional and taxonomic information based on ORF homology	−	−	Wommack et al., 2012
VirSorter	Homology	Gene homology including enrichment of viral-like and short genes, and depletion of PFAM hits and strand shifts	NCBI RefSeq genomes before January 2014 and environmental viromes.	wget	Roux et al., 2015
VirSorter2 (2.2)	Homology	Random forest classifiers using an hmmsearch of genes predicted with Prodigal	NCBI RefSeq genomes before January 2020 and high-quality genomes from the literature.	Bioconda	Guo et al., 2021
VirusSeeker	Homology	BLAST search against virus database, followed by search against full NCBI database to remove false positives	Viral-only NCBI NT and NR database before August 2016	−	Zhao G. et al., 2017

### 2.2. Datasets

This study leverages 4 benchmark datasets: (1) *genome fragment set* used for assessing the effect of contig length, low viral abundance, eukaryotic contamination, and potential bias toward certain groups of phages, (2) *simulated phageome set* used to explore the effect of sequencing error on the classification, (3) *simulated metagenome set*, used for exploring the effect of the quality of assembly, and viral abundance in samples. The study also includes a real gut metagenome dataset from colorectal cancer (CRC) patients compared to healthy controls: (4) *CRC dataset* used to compare the results of the tools on a real metagenomic dataset. Finally, this study includes real gut viromes from children with Crohn’s disease, ulcerative colitis, and healthy controls: (5) *gut virome dataset*.

#### 2.2.1. Set 1: Genome fragment set

All complete bacterial, fungal, and viral genomes were downloaded from the RefSeq database on 14 June 2021 (O’Leary et al., 2016). These genomes were fragmented into non-overlapping adjacent fragments of lengths 500, 1,000, 3,000, and 5,000 nucleotides. In total, 379 archaeal, 21,788 bacterial, 18 fungal, and 11,156 viral genomes were obtained and fragmented, of which 1,483 were phage. From those fragmented genomes, 10,000 fragments were randomly selected from each length and each superkingdom. The resulting set includes four subsets: 500, 1,000, 3,000, and 5,000 bp, each with 10k fragments from the four superkingdoms for a total of 40k fragments per length subset. This collection of unmodified fragmented reference genomes is referred to as the *genome fragment set*.

#### 2.2.2. Set 2: Simulated phageome set

InSilicoSeq v 1.5.4 (Gourlé et al., 2019) was used for creating simulated reads from phage genomes. This tool creates an error model of per-base quality (Phred) scores using Kernel Density Estimation, trained on real sequencing reads. InSilicoSeq was chosen due to its computational efficiency (McElroy et al., 2012), simplicity of use and documentation (Yu et al., 2020), and ability to simulate Illumina sequencing instead of 454 technology (Richter et al., 2008; Zhao M. et al., 2017). Additionally, this tool has been demonstrated to generate reads with realistic quality score distributions for several sequencing platforms, including MiSeq, HiSeq, and NovaSeq (Gourlé et al., 2019; Yu et al., 2020).

Three “phageome” profiles were created by randomly selecting 500 phage genomes per profile from the downloaded RefSeq database. Reads were simulated using InSilicoSeq, specifying 30x coverage of all genomes. Reads simulated using each of the three built-in error models were created for each profile. Simulated reads from InSilicoSeq were assembled with MEGAHIT v1.2.9 (Li et al., 2015) and binned with MetaBAT 2 v2:2.15 and using Bowtie2 v2.4.5 for indexing (Langmead and Salzberg, 2012; Kang et al., 2019).

To determine the genomic origin of each contig, BLAST v2.12.0+ was used for alignment. Since the genomes used for read simulation for each profile were known, a local BLAST database was created using those same genomes for each simulated phageome, reducing spurious hits. Alignment was done using the MEGABLAST mode of BLASTn, with an *e*-value of 1e-20. Even with the limited BLAST databases, it was common for contigs to have significant hits to several genomes. To determine the “true” origin of the contigs, a basic decision tree was used which is shown in Supplementary Figure 3.

In total, the *simulated phageome set* is comprised of nine phageome assemblies and bin sets (3 profiles * 3 error models).

#### 2.2.3. Set 3: Simulated metagenome set

The same simulation and binning steps used in the Set 2 were used to generate a set of simulated metagenomes. Five marine samples were used as the basis of this dataset. Three were from the Hawaii Ocean Time-series (HOT) program (SRR5720259, SRR5720320, SRR6507280) (Karl and Lukas, 1996), and two were from the Amazon continuum dataset (SRR4831655, SRR4831664) (Satinsky et al., 2014). Raw sequencing data were downloaded from Sequence Read Archive (SRA), and processed using fastqc v0.11.9 and trimGalore v0.6.6 (Andrews, 2010; Babraham Bioinformatics, 2022). Briefly, reads with average base quality score below 20 were removed, and those with adapters and poly-G sequences were trimmed. After trimming, reads with a length < 20 bp were filtered out. After quality control (QC), taxonomic abundance profiles of the bacterial and phage population in each sample were obtained using Kraken2 (Wood et al., 2019) and Bracken (Lu et al., 2017) against the PlusPF database (version 5/17/2021 available at https://benlangmead.github.io/aws-indexes/k2). The abundance profiles were used as input for InSilicoSeq, using reference genomes obtained from the RefSeq database. Additionally, for any profile with a phage abundance below 5% of reads, the profile was supplemented with additional phages by adding a minimum of 10 phages known to infect the top non-viral organisms in the profile. 20M Simulated reads for each profile were generated using the three built-in error models (HiSeq, MiSeq, and NovaSeq). The 15 resulting assemblies and bins are referred to as the *simulated metagenome set*.

#### 2.2.4. Set 4: CRC dataset and Set 5: Gut virome dataset

We also included a real-metagenomic dataset from a published study that used fecal shotgun metagenomics to characterize stool microbial populations from CRC patients compared to healthy controls with a total of 198 samples (Zeller et al., 2014). This dataset is referred to as the *CRC dataset* in this study. Additionally, we included a real virome dataset from a previously published study that used viral particle enrichment on fecal samples (Fernandes et al., 2019) from 24 healthy and IBD children. This second dataset is referred to as *gut virome dataset* in this study.

Raw sequencing data were downloaded from SRA (PRJEB6070 and PRJNA391511) and were quality filtered using fastqc v0.11.9 and trimGalore v0.6.6. Briefly, reads with an average base pair quality score below 20 were removed, and adapters and poly-G sequences were trimmed. After trimming, reads with a length < 20 bp were filtered out. Quality-filtered sequences were screened to remove human sequences using bowtie2 v2.4.2 against a non-redundant version of the Genome Reference Consortium Human Build 38, patch release 7 (available at PRJNA31257 in NCBI).

After QC and human read filtering, the reads were assembled using Megahit v1.2.9. The code of the pipeline used for the assembly is available on Github.^1^ Megahit was run on the paired-end reads or single-end reads using the default parameters (referred to as the simple assembly). Additionally, a co-assembly of the multiple runs per BioSample was also performed (referred to as the co-assembly). Assemblies were binned with MetaBAT 2 v2:2.15 and using Bowtie2 v2.4.5 for indexing. CheckV v1.0.1 was run on all assemblies to assess viral and bacterial gene content.

### 2.3. Classification of the datasets

Snakemake was used as a workflow manager for running the tools (Köster and Rahmann, 2012). This pipeline was implemented on the Puma High-Performance Compute (HPC) cluster at the University of Arizona using SLURM (Yoo et al., 2003). While running the tools, the following metrics were collected by Snakemake: runtime and CPU time, peak memory usage, and file write operations.

When running the tools, the default parameters, modes, and databases were used to replicate those intended for use by the authors. DeepVirFinder was run without a length cutoff. MetaPhinder was run using the default database. Seeker was run using the command-line executable binary instead of the Python package. VIBRANT was run in standard (not virome) mode, with the default minimum length (1,000 bp) and number of ORFs (4). viralVerify utilized the default database. Virsorter was run using the default (RefSeq) database, in non-virome mode, and BLASTP as the default was used instead of DIAMOND. Virsorter2 was run to identify only dsDNAphage and ssDNAphage, allowing for proviruses by not using the “–no-pro-virus” flag and not limiting the number of ORFs.

All of the tools classified the fragments in the *genome fragment set* except for MARVEL, which requires bins as input. VIBRANT and VirSorter do not classify fragments shorter than 1,000 nucleotides (nt), so there is no data for these tools for the 500 nt fragments. Default parameters were used for cutoff thresholds when the option was provided. To simplify the comparison of the classification performances, all predictions were binned into “phage” or “non-phage” classes. VirFinder and DeepVirFinder return a prediction score, and a score of 0.5 was used as a cutoff for classification. VIBRANT predicts both prophage and lytic virus labels, both of which were considered to be classified as phage. VirSorter also predicts prophage and lytic labels, and assigns a confidence category, all of which were considered to be classified as phage.

The simulated datasets (*simulated phageome set* and *simulated metagenome set*), and the *CRC dataset*, and the *gut virome dataset* were classified by all tools. Assembled reads were classified by all tools except MARVEL. MARVEL was given binned assemblies for classification. Resource usage required for binning was included in the resource usage benchmarking for MARVEL.

### 2.4. Performance assessment

Several performance metrics are assessed for each of the challenge datasets. These are precision, sensitivity (recall), specificity, *F*1, and AUPRC, as defined below. In these definitions, a “positive” is a phage sequence, while a negative is anything that is not phage. Accordingly, a true positive (TP) is a phage sequence that has been correctly labeled as phage, a True Negative (TN) is a non-phage sequence correctly labeled as such, a False Positive (FP) is a non-phage sequence labeled as phage, and a False Negative (FN) is a phage sequence not labeled as phage. Precision is the portion of all predicted phage sequences that are indeed phage (Eq. 1). Sensitivity, also known as recall, is the proportion of all true phage sequences that were correctly identified (Eq. 2). Specificity is the proportion of all non-phage sequences that were correctly labeled (Eq. 3). *F*1 score is the harmonic mean of precision and sensitivity (Eq. 4). The area under the precision recall curve (AUPRC) is a measure of precision over the sensitivity range, given a varying classification threshold for a continuous predictive output value (Eq. 5).


(1) $$p\,r\,e\,c\,i\,s\,i\,o\,n=\frac{T\,P}{T\,P+F\,P}$$

(2) $$s\,e\,n\,s\,i\,t\,i\,v\,i\,t\,y\,\left(r\,e\,c\,a\,l\,l\right)=\frac{T\,P}{T\,P+F\,N}$$

(3) $$s\,p\,e\,c\,i\,f\,i\,c\,i\,t\,y=\frac{T\,N}{T\,N+F\,P}$$

(4) $$F\,1=\frac{2\times p\,r\,e\,c\,i\,s\,i\,o\,n\times s\,e\,n\,s\,i\,t\,i\,v\,i\,t\,y}{p\,r\,e\,c\,i\,s\,i\,o\,n+s\,e\,n\,s\,i\,t\,i\,v\,i\,t\,y}$$

(5) $$A\,U\,P\,R\,C=\int_{0}^{1}p\,dr;p=p\,r\,e\,c\,i\,s\,i\,o\,n,r=r\,e\,c\,a\,l\,l$$

## 3. Results

### 3.1. Installation of tools

A total of 19 tools were collected based on a survey of the literature as of July 2021. However, several tools were omitted from further investigation for the following reasons: (1) the creation of runtime exceptions (PhaMers and VirMine), (2) tools with hard-coded paths that require the user to modify source code (RNN-VirSeeker and VirusSeeker), (3) lack of clear installation instructions and documentation (ViraMiner and VirNet), (4) tools that are unscalable due to web server usage (VirMiner and VIROME), and (5) inability to run instances of the tool on different cores from the same directory (PPR-Meta). Finally, Cenote Unlimited Breadsticks could be installed and run but did not classify any of the genome fragments as viral, and was excluded from the benchmark analysis, but included in the resource usage comparison.

### 3.2. Resource usage

Computational resource usage was benchmarked using the *genome fragment set* since the quantity and length of genomic fragments were known and balanced. Pre- and post-processing steps were excluded from these measurements. For tools that did not allow the user to specify the output directory (MARVEL and Seeker), we also included time to move output files to the correct output directory.

**FIGURE 1:**
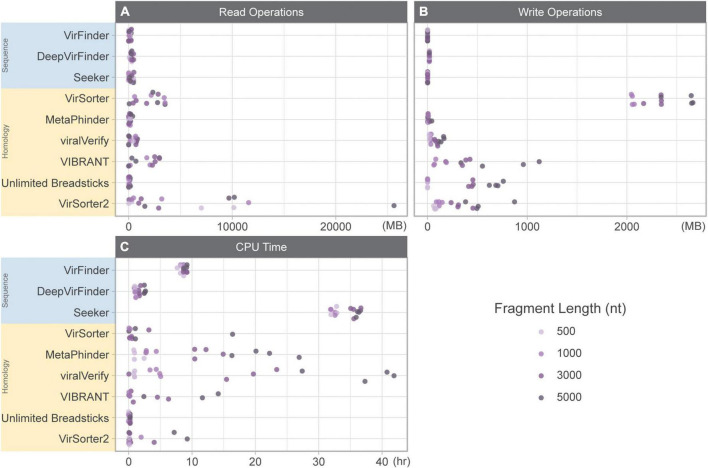
Resource usage while classifying 10k genome fragments of various lengths (500, 1,000, 3,000, and 5,000 nt). **(A)** Read operations (MB), **(B)** write operations (MB), and **(C)** CPU time (h) summed for user and system. Sequence-based tools are in blue, homology-based tools are in yellow.

The total time (in CPU time) for each tool included: (1) CPU time to run the tool summed for user and system and (2) the amount of time to read and write data while classifying the *genome fragment set* (Figure 1). For some tools, CPU time was highly variable (MetaPhinder, VIBRANT, viralVerify, and to a lesser extent for VirSorter, VirSorter2, and Seeker). Seeker generally had the longest CPU times, even for shorter fragments. While DeepVirFinder was consistently fast, its real-world performance was hindered due to its use of the Theano backend. While multiple jobs can be submitted in parallel, the Theano backend can only process one dataset at a time for serial processing. This led to long-running jobs for DeepVirFinder, but deceptively low CPU time measures.

### 3.3. Benchmark challenge 1: Classification of genome fragments

#### 3.3.1. Effect of contig length

**FIGURE 2:**
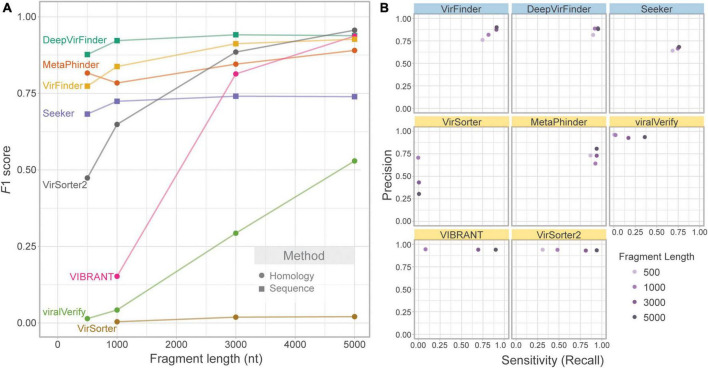
Effect of fragment length on classification performance. Only phage and bacterial sequence fragments are included. **(A)** Balanced *F*1 score plotted against fragment length (nt) and **(B)** balanced precision plotted against sensitivity for four fragment lengths. Top row of tools are sequence-based (in blue), bottom two rows are homology-based (in yellow).

We first evaluated the effect of contig length on each tool’s performance. To assess this effect, the *genome fragment set (Set 1)* was used as input. Figure 2A shows *F*1 score for increasing fragment lengths (500, 1,000, 3,000, and 5,000 nt). As expected, homology-based tools such as VIBRANT, viralVerify, and VirSorter2 were strongly affected by fragment length, with performance increasing with length. VirSorter had the lowest *F*1 score for all lengths and had only a marginal increase in *F*1 with increasing length. The sequence-based tools (DeepVirFinder, VirFinder, and Seeker), as well as MetaPhinder, were largely unaffected by fragment length.

While *F*1 score illustrates overall changes in classification performance due to fragment length, each tool’s performance is affected in different ways (Figure 2B). VIBRANT, viralVerify, and VirSorter2 demonstrate fairly consistent and high precision but have length-dependent sensitivity, whereas DeepVirFinder, MetaPhinder, Seeker, and VirFinder demonstrate fairly consistent sensitivity and precision. Generally, length-dependent sensitivity is a property of homology-based tools where longer fragments are needed for classification. Interestingly, MetaPhinder (a homology-based tool) exhibits a pattern similar to the sequence-based tools for this property.

Several tools output a continuous classification score metric. For these tools (VirFinder, DeepVirFinder, Seeker, MetaPhinder, and viralVerify), the threshold used will affect precision and sensitivity. Although the default threshold was used, the effect of this threshold can be seen in the precision-recall curves (Supplementary Figure 1) and AUPRC (Supplementary Figure 2). These tools all showed lower AUPRC for shorter contigs, with DeepVirFinder outperforming the other tools even on shorter contigs.

#### 3.3.2. Low viral content

**FIGURE 3:**
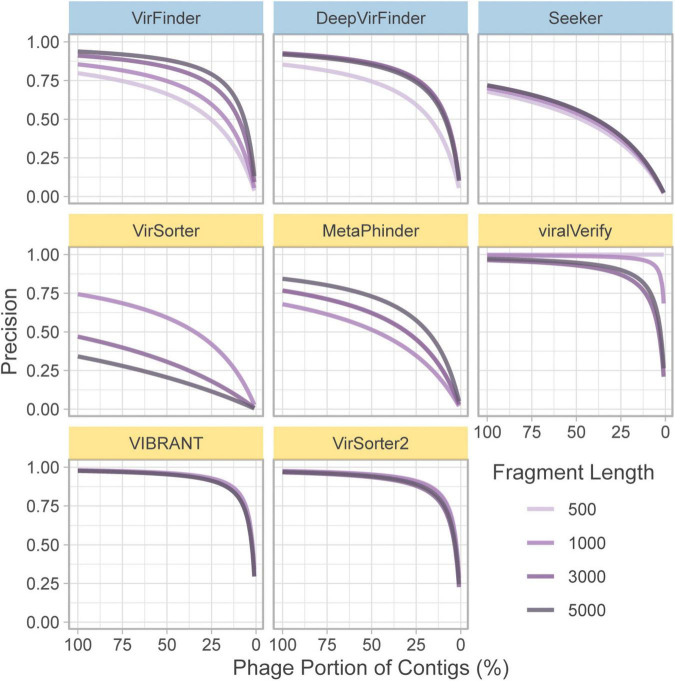
Extrapolated precision calculated from FPR of each tool at four fragment lengths (500, 1,000, 3,000, and 5,000 nt). Precision is calculated for communities composed of varying levels of non-viral fragments from 0% (all phage) to 100% (all non-phage). Top row of tools are sequence-based (in blue), bottom two rows are homology-based (in yellow).

In the above section, precision is computed based on a balanced dataset (equal quantities of phage and bacteria). However, this gives a highly optimistic estimate of precision. For a given false positive rate (FPR), precision will drop significantly when phage content is low. To illustrate this, the FPR (Eq. 6) was taken from the classification of the *genome fragment set*, and precision was extrapolated to hypothetical community compositions ranging from 0 to 100% non-viral fragments (Figure 3). 50% represents a balanced dataset.


(6) $$F\,P\,R=\frac{F\,P}{F\,P+T\,N}$$

For communities with low viral content, precision decreases for nearly all tools. Notably, viralVerify did not falsely classify any 500 nt fragments as phage, thus its hypothetical precision remains perfect for that fragment length. VIBRANT, viralVerify, and VirSorter2 maintain fairly high precision but still drop below 0.5 for communities with low viral content.

#### 3.3.3. Effect of phage taxonomy

**FIGURE 4:**
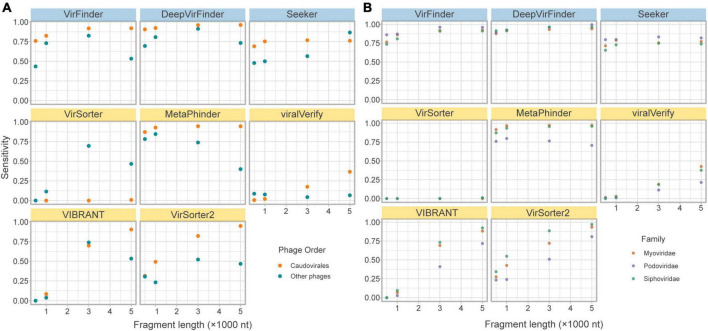
Effects of phage taxonomy on sensitivity. **(A)** Sensitivity plotted against fragment length, comparing bacteriophages in the order Caudovirales and those in other orders. **(B)** Sensitivity plotted against fragment length, comparing bacteriophages in the top three families of the order Caudovirales. Top row of tools are sequence-based (in blue), bottom two rows are homology-based (in yellow).

The lack of phage diversity and bias for certain phage groups in reference databases leads to challenges in training models and propensity for tools to retrieve fewer phages from less represented phage groups. The majority (c.a. 93%) of phages in RefSeq belong to the order Caudovirales (recently renamed as the class Caudoviricetes; Turner et al., 2021). Importantly, all tools were shown to have reduced sensitivity for non-caudovirales sequences. In particular homology-based tools showed a drastically reduced sensitivity toward these phages (Figure 4).

While the limitation of database composition is mitigated by sequence-based compared to homology-based approaches, a slightly lower sensitivity for non-caudoviral phages was nonetheless observed.

Even within the caudoviral order, the three main families are detected unequally by the tools. *Siphoviridae* constitutes the greatest phage family represented in RefSeq (69.9%), followed by *Myoviridae* (15.6%) and *Podoviridae* (7.28%). This is apparent in the *fragmented genomes set* due to random sampling from the fragmented genomes; of the caudoviruses, more fragments came from *Siphoviridae* and *Myoviridae* than *Podoviridae*. Figure 4 demonstrates how sensitivity is decreased for the retrieval of *Siphoviridae* and *Podoviridae* sequences compared to *Myoviridae* in particular MetaPhinder, VIBRANT, and VirSorter2.

#### 3.3.4. Eukaryotic contamination

**FIGURE 5:**
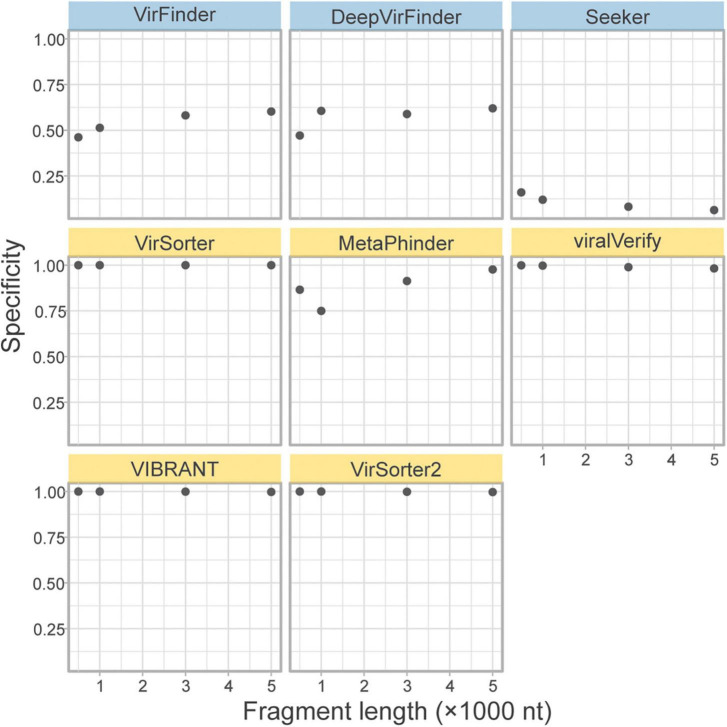
Classification specificity of eukaryotic genome fragments. Eukaryotic fragments were generated from the Ascomycota and Basidiomycota phyla (*n* = 10k) at different length size. The specificity of each tool was measures for each sequence length. Top row of tools are sequence-based (in blue), bottom two rows are homology-based (in yellow).

A concern for sequence-based tools is specificity when faced with eukaryotic contamination, due to the lack of eukaryotic sequences in the training sets (Ponsero and Hurwitz, 2019). As part of the *genome fragment set*, the tools classified 10k eukaryotic genome fragments of 4 lengths from fungi in the phyla Ascomycota and Basidiomycota. The specificity on these eukaryotic fragments is shown in Figure 5. All homology-based tools are extremely robust to eukaryotic contamination even for short fragments. However, sequence-based tools and Metaphinder show much lower specificity, frequently misclassifying eukaryotic fragments as viral, with an FPR around 0.5. Notably, Seeker shows a sensitivity that is worse than a random chance binary classification, classifying nearly all eukaryotic sequences as viral.

### 3.4. Benchmark challenge 2: Classification of simulated metagenomic sequences

To compare the relative performance of the tools when faced with read errors from different sequencing technologies and potential assembly error, the *simulated phageome set* and *simulated metagenome set* were created from simulated reads generated using error models that represent 3 popular sequencing platforms: HiSeq, MiSeq, and NovaSeq. Each technology has a unique per-base error rate, as modeled by InSilicoSeq, as well as differing read lengths. We aimed to assess how these differences affect assembly and tool performance.

#### 3.4.1. Simulated phageomes

**FIGURE 6:**
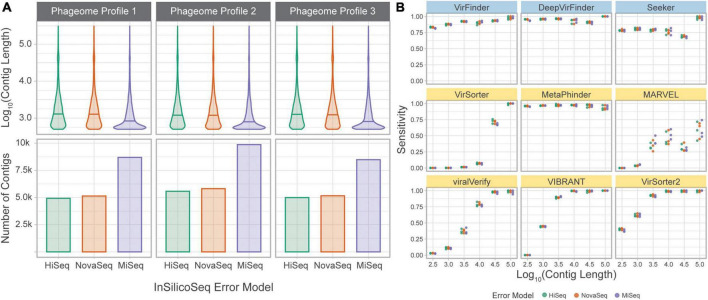
Tools performances on a simulated phageome dataset. **(A)** Length distributions, and number of, assembled contigs constituting the nine phageomes generated using the three phageome profiles and three error models. **(B)** Sensitivity of classifying simulated phage contigs. Contigs were grouped by length (*x*-axis) for computation of sensitivity. Error models are ordered by increasing read length. Top row of tools are sequence-based (in blue), bottom two rows are homology-based (in yellow).

To directly assess the effect of sequencing error and assembly on classification sensitivity, the *simulated phageome set* was classified by each tool. In this dataset, simulated reads were obtained from phage genomes and assembled into contigs. The assembled contigs’ length varied from 500 to 309,196 bp, with a median length of 949 bp. Unlike in the *genome fragment set*, simulated contigs could be used to assess MARVEL, which requires binned sequences for classification. Each tool’s sensitivity was calculated using the assembled contigs grouped by length (Figure 6).

The results are largely consistent with those obtained using the *genome fragment set*. DeepVirFinder and MetaPhinder, followed by VirFinder and Seeker, show the highest and most consistent sensitivity across all contig lengths. VIBRANT, VirSorter, and VirSorter2 performed well for longer contigs, but sensitivity suffers as contig length decreases. VirSorter’s sensitivity begins to improve at about 10^4^ bp and increases greatly for 10^4.5^ and 10^5^ bp. MARVEL, while better than VirSorter on short contigs, demonstrates the lowest sensitivity for long contigs. Importantly, MARVEL shows more variability in sensitivity for a given length. Indeed, the tool performs classification of contigs after binning, and since contigs of various lengths may be present in the same bin, we observe that the tool’s performance is less tightly coupled to contig length.

The three error models produce reads of different lengths (HiSeq 125 bp, NovaSeq 150 bp, and MiSeq 300 bp). This led to a significant difference in contig lengths between MiSeq and the other two error models (based on Wilcoxon rank sum test with Bonferroni adjusted *p*-value, *p* < 0.05). However, the sensitivity at a given length was similar across error models, suggesting that the difference in sequencing technologies is mitigated by the assembly process.

#### 3.4.2. Simulated metagenomes

The *simulated metagenome set* was produced from the bacterial and phage content of 5 metagenomic marine samples. This method allowed us to generate simulated metagenomes that are as close and possible to a real metagenome set while excluding the unknown fraction of the microbial population. The distance between the original taxonomic profile for the sample and the profile used for simulation was calculated as Bray-Curtis dissimilarity (Supplementary Figure 4). This computational method allowed us to generate simulated metagenomes containing 5% of phage sequence content and a realistic distribution of contigs length.

**FIGURE 7:**
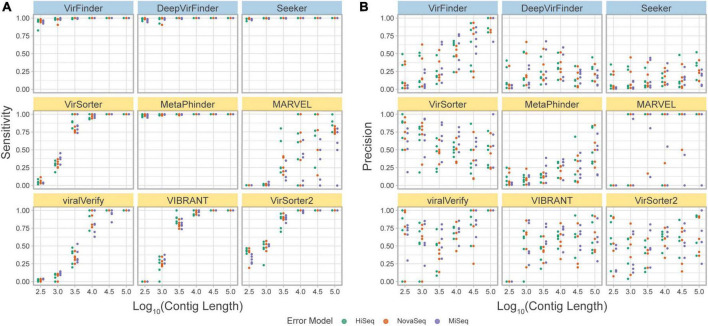
**(A)** Precision and **(B)** sensitivity on a simulated metagenome dataset. Each point represents the tool’s performance on contigs within a given length group, assembled from reads from a specific abundance profile, using the indicated error model. Top row of tools are sequence-based (in blue), bottom two rows are homology-based (in yellow).

The precision and sensitivity of the tools on the *simulated metagenome set* were assessed based on the contig length (Figure 7, and *F*1 score in Supplementary Figure 4). The observed sensitivity (Figure 7A) is consistent with the results from the *simulated phageome set*, although MARVEL displays a large variance in sensitivity across the different replicates, possibly reflecting the quality of binning.

Importantly, using this benchmark set, a decrease in precision due to low viral abundance is clearly seen for all tools (Figure 7B). VirSorter, viralVerify, VIBRANT, and VirSorter2 perform slightly better than VirFinder, Seeker, and MetaPhinder. viralVerify performs exceptionally well for long contigs (≥10^5^), followed closely by VirFinder.

### 3.5. Benchmark challenge 3: Comparison on a real-world dataset

We finally compared the tools on two real datasets, the *CRC dataset* and the *gut virome dataset*, for which the true phage/bacterial composition is unknown. We aimed here to assess the overlap in phage identification of the different tools and estimate the number of potential FP results.

#### 3.5.1. CRC dataset

Importantly, when comparing the results obtained for tools on the CRC dataset the proportions of contigs predicted to be phage vary strongly by tool. VirSorter, MARVEL, viralVerify, VIBRANT, and VirSorter2 detect fewer phages (median less than 2,200 contigs per sample) than the sequence-based methods and MetaPhinder (median greater than 33,000 contigs per sample). This result is consistent with the high precision but lower sensitivity measured for homology-based tools.

**FIGURE 8:**
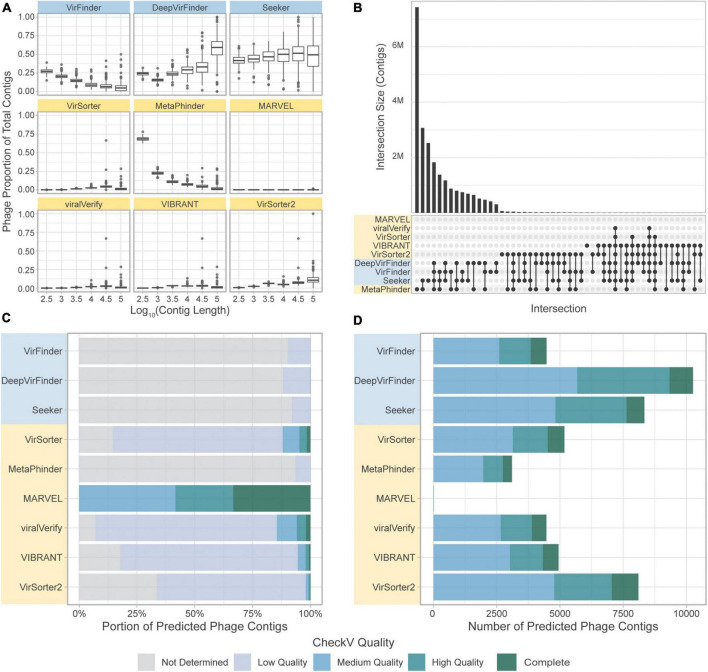
Comparison of the tools’ classifications on a real-world metagenomic dataset. **(A)** Proportion of contigs predicted to be phage in each length group for each sample. **(B)** Upset plot showing intersection size, with the *x*-axis in order of decreasing set size, top 51 intersections shown. **(C)** CheckV assessment of predicted phage contigs from the *CRC dataset*. Predicted phage contigs from each tool are categorized by CheckV, and plotted as a stacked bar chart of the portion of predicted phages in each category. **(D)** Total number of contigs predicted by CheckV to have quality Medium or greater. Sequence-based tools are in blue, homology-based tools are in yellow.

We next assessed the overlap of the different tools in predicting the same sequence as phage (Figure 8). Strikingly, there was very little overlap in phage communities predicted by the tools (Figure 8B). The highest level of consistency between tools was seen for VirFinder and DeepVirFinder (38.8% of contigs identified by either tool were identified by both), and the highest level of consistency between homology-based tools was with VirSorter and VIBRANT (26.6% of predicted phages were in common). However, most contigs showed different levels of consistency between tools, where on average, 55,320 contigs were predicted to be phage by only one tool, 62,400 were predicted by 2 or more tools, and 29,900 by 3 or more tools.

CheckV (Nayfach et al., 2021a) was used to evaluate the predicted phage contigs for viral genes. Potential viral contigs are categorized as “Not Determined” (without any detectable viral genes), “Low Quality,” “Medium Quality,” “High Quality,” or “Complete.” The proportions of contigs predicted by the tools falling into each category are shown in Figure 8C. As expected, the sequence-based tools and MetaPhinder contain a low proportion of contigs with Medium Quality or higher, suggesting a potential number of FPs for these tools, while the other homology-based tools have higher quality predictions. MARVEL predicted no contigs below Medium Quality. Additionally, the number of predicted phages being labeled as Medium Quality, High Quality, or Complete by CheckV are shown in Figure 8D.

#### 3.5.2. Gut virome dataset

**FIGURE 9:**
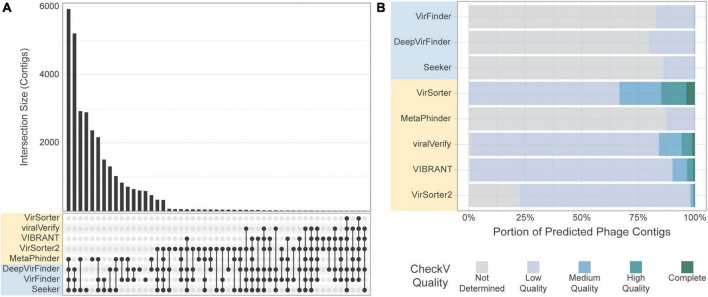
Comparison of the tools’ classification on real-world viromes. **(A)** Upset plot showing intersection size, with the *x*-axis in order of decreasing set size, top 51 intersections shown. **(B)** CheckV assessment of predicted phage contigs from the Gut Virome dataset. Predicted phage contigs from each tool are categorized by CheckV, and plotted as a stacked bar chart of the portion of predicted phages in each category.

When comparing the results obtained for each tool on the *gut virome dataset*, we observe that, similarly to the *CRC dataset*, the quantity of contigs predicted as viral varies strongly by tool. However, we observe an increased consistency among the tools, and the phage communities retrieved by the tools had a larger overlap (Figure 9A). Similar to the *CRC dataset*, the highest level of consistency between tools was seen for VirFinder and DeepVirFinder (60.5% of contigs found in common), and the consistency between homology-based tools remained low, with the greatest agreement found between VIBRANT and viralVerify (22.8% of predicted phage sequences in common).

Most sequences retrieved by sequence-based tools were still classified as “Not determined” by CheckV, suggesting a potential high number of FPs for these tools in these conditions (Figure 9B). Interestingly, the number of contigs classified as “Medium quality” or higher by CheckV was similar among tools, with the exception of Metaphinder and VirSorter which retrieved fewer contigs (Supplementary Figure 8).

### 3.6. Reusable benchmark dataset

The five benchmark datasets are available on Zenodo (doi.org/10.5281/zenodo.7194616). Files for the *genome fragment set* include a FASTA file of genome fragments, a CSV file of taxonomic information of each fragment, and a compiled and cleaned file of the classification results of the nine tools on the genome fragments. Files for the *simulated metagenome set* and *simulated phageome set* include the assembly files in FASTA format, the binned assemblies, a CSV file of the contig taxonomic origins resulting from BLAST, and a CSV file of the compiled contig classifications by the tools. For the *CRC dataset* and *gut virome dataset*, the assemblies and bins, as well as the compiled contig classifications, are available. For all datasets, the resource usage as recorded by Snakemake is also included.

In addition to README files for each dataset, datasheets based on Datasheets for Datasets (Gebru et al., 2021) are deposited in Zenodo. These provide details about how the datasets were generated, their composition, and the intended uses.

## 4. Discussion

This study aims to gain a better understanding of how metagenomic phage detection tools perform under a variety of conditions. Previous studies have explored the detection and classification of dsDNA viruses and auxiliary metabolic genes, but the performance of these tools under a variety of challenges remains. Given differences in metagenomes based on the taxonomic composition, sequencing quality, and computational methods for analyzing these metagenomes, we examined the effects of contig length, phage taxonomy, and sequencing and assembly error. We sought to address the robustness of tools to eukaryotic contamination and low viral content. Finally, we wanted to address the different phage communities predicted by the tools when classifying real gut metagenomes and viromes.

### 4.1. Tools installation, reusability, and computational requirements

One of the first barriers to the effectiveness of a phage detection tool, and bioinformatics tools in general, is the ability to be installed and scaled to real data. Of the 19 tools identified for this study, only 9 (47%) could be installed and run at scale. This is corroborated by related studies, such as Pratama et al. (2021), which excluded PHASTER and VirMiner, and Ho et al. (2021), which excluded VIROME, VirMiner, ViraMiner, PhaMers, VirNet, and VirMine from their benchmarking efforts. Tools that were available on Bioconda and PyPi were easiest to install because the tool and all dependencies could be installed simultaneously, while those on GitHub required more effort. We acknowledge the extra effort required to develop tools and create releases using Bioconda and PyPi.

There have been efforts to make tools more accessible, even if the original version is not so straightforward to install. One solution is sharing containers, such as on DockerHub, Biocontainers on the AWS ECR public gallery, or CyVerse (da Veiga Leprevost et al., 2017; CyVerse, 2022; Docker Hub, 2022). One effort specific to phage-finding tools is What the Phage, which developed a Nextflow-based workflow for running several phage detection tools in a single container (Marquet et al., 2022).

In addition to the ability to install and run a tool, scalability and resource usage are significant factors considering the scale of metagenomic data. While web services provide a convenient interface, they are often not a viable option for classifying many samples. Similarly, tools that do not allow several samples to be processed at the same are impractical in most cases. For the tools that could be installed and run at scale, computational resource usage varied widely. Homology-based tool compute times generally varied with contig length. Seeker had the most consistently high compute times (greater than 30 h to classify 10k fragments). While DeepVirFinder had the most consistently low compute times, its inability to process several samples in parallel hindered its actual runtimes. However, this may be circumvented by running each process in its own container, each with its own Theano backend.

### 4.2. Performance on short sequences

In this study, we compared the tools on several challenges to identify the current limitations and strengths of tools and computational approaches. First, we evaluated the effect of sequence length on classification performance. Unsurprisingly, sequence-based tools were able to work with shorter contigs than homology-based tools, as they did not require the presence of multiple genes to classify a sequence. Similarly, the precision of homology-based tools was largely independent of fragment length since shorter contigs would not be expected to affect the FPR. Finally, the sensitivity of sequence-based tools such as VirFinder, DeepVirFinder, and Seeker were globally unaffected by the sequence length and allow the retrieval of sequences as short as 500 bp. However, a decreased precision for shorter contigs could be observed for these tools, suggesting that a higher number of FPs should be expected when using these tools on shorter contigs. On the other hand, homology-based tools such as VirSorter2, Vibrant, and viralVerify, showed a reduced sensitivity on short contigs but a consistent precision for all lengths. Interestingly, Metaphinder, which leverages multiple hits against a genome database, showed a similar effect for contig length as the sequence-based tools, with an increased FPR for shorter contigs. Metaphinder sums the regions of the fragment that have BLASTn hits to phage genomes in a reference database, even if the hits are from distinct phage groups. This, coupled with a fairly permissive *e*-value of 0.05, leads to a sensitive but less precise classification for short fragments.

The result of this reduced sensitivity on short contigs is seen in both the *simulated phageome set* and *simulated metagenome set*, where VirSorter, viralVerify, VIBRANT, and VirSorter2 recover less than 75% of phage contigs between 1,000 and ∼3,200 bp long. Considering the difficulty of assembling short reads in real metagenomes, this poses a barrier to retrieving a large number of phage sequences present only as short contigs. For perspective, 93% of the contigs in the *CRC dataset* were shorter than 3 kbp.

### 4.3. Bias toward over-represented phage groups

The ability of these tools to identify novel phages or phages with lower database representation can be critical for exploring many natural viral populations. We assessed this by comparing the sensitivity of the tools on the well-represented Caudoviral group and other phage groups. Importantly, all tools showed a decreased sensitivity for non-caudoviral phages. This effect was also seen when comparing the sensitivity of tools on the more abundant Myoviridae phages compared to the Siphoviridae and Podoviridae phages. This result is particularly striking as it suggests a bias in sensitivity toward the over-represented phage groups in databases. Of the tools included in this benchmark, DeepVirFinder appears to be the most suited to detecting a wider diversity of phages and showed the most consistent sensitivity across phage groups. It is important to note here that our benchmark dataset relied on RefSeq genome sequences and is therefore limited to known phages.

### 4.4. Low viral content and eukaryotic sequences

Real metagenomes typically contain low levels of viral content and may also carry eukaryotic sequences from the host (e.g., human gut microbiome) or from micro-eukaryotes. While sequences from a eukaryotic host can typically be excluded from the metagenomic dataset before viral detection, this is particularly difficult for micro-eukaryotic sequences. Of the tools compared, none used eukaryotic genomes in their training set, leading to concerns about specificity when faced with eukaryotic contamination. In this benchmark, we showed that sequence-based tools and Metaphinder exhibit low specificity on fungal sequence fragments, while other homology-based tools remain unscathed.

To understand how low viral content affects precision, the precision of tools was extrapolated to the full range of possible phage content. All tools had decreased precision when viral content is low, dropping sharply when viral content is below 20%. viralVerify was the most robust to low viral content, especially on shorter contigs. The consequences of this were seen in the classification of *simulated metagenome* set, each of which had 5% phage. All tools had varying and often low (below 0.5) precision, although viralVerify and VirFinder had good precision for the longest contigs.

### 4.5. Sequencing error and assembly quality

Using simulated datasets, we next aimed to assess the effect of sequencing error and assembly on each tool’s performance. This method allowed us to develop more realistic benchmark sets while retaining the possibility to assess the true composition of the set.

First, we evaluated the tools’ sensitivity on a *simulated phageome set* composed of simulated phage contigs only and assessing the potential effect of sequencing error and potential misassembly. We showed the global sensitivity to be very similar to that obtained when classifying unmodified genome fragments. This indicates that sequencing error, sequencing technology, and misassembly do not hinder sensitivity significantly, at least with sufficient sequencing depth (simulated phageomes were simulated with 30x coverage).

The *simulated metagenome set* aimed to give the most realistic estimate of real-world performance. The sensitivity of all tools again closely reflected previous results. In particular, the contig length and the low viral content were driving the observed tools’ performances. DeepVirFinder, Seeker, and at shorter contig lengths Metaphinder, had precision typically below 0.5. VirSorter, viralVerify, VIBRANT, and VirSorter2 had slightly higher precision, although with high variance, often falling below 0.5. viralVerify, however, was extremely precise for long contigs. Once again, this result suggested a limited effect of sequencing error on the tools’ performances.

### 4.6. Comparing overlap in viral predictions

The previous benchmark challenges suggested vastly different properties that affect the final result obtained by users when using on their real-world datasets. This was further demonstrated here on the *CRC dataset*. The predicted phage quantity and composition is strikingly different between tools. As expected from the previous benchmarks, the homology-based tools, excluding MetaPhinder, predict far fewer phages than sequence-based tools. But most strikingly, the overlap of sequences found by several tools is surprisingly low. Of all sequences predicted to be phage by at least one tool, only 53% were predicted by two or more tools, and 25% were found by three or more tools, on average. The dissimilarity of contigs predicted as phage by the tools is so wide that approximately 80% of contigs are predicted to be phage by at least one tool. Consequently, when applying these tools to real datasets, the choice of tool would strongly affect the predicted phage community.

The use of CheckV can help reach a larger level of agreement between tools, when used to filter out potential FPs (Figure 8D and Supplementary Figure 7). In CheckV, genes are first annotated as either viral or microbial based on comparison to a large database of hidden Markov models (HMMs), and the absence of detectable viral genes in the sequence leads to a classification of the contig as “Not determined.” Interestingly, when contigs with Low Quality or Not Determined status are removed, then greater than 50% of contigs from the *CRC dataset* found by at least one tool are found by 3 or more tools; 26% of contigs found by at least one tool are found by 6 or more tools. However, CheckV is stringent, and additional contigs can be recovered by supplementing the dataset with contigs that lack genes of cellular origin, especially for metagenomes with highly novel phage.

For viral-particles enriched metagenomes (*gut virome dataset*), the results obtained from the different tools were more consistent. With fewer non-viral sequences, the number of FPs should be reduced in particular for the sequence-based tools, explaining a more consistent results among the tools.

### 4.7. Toward a reusable benchmark dataset for viral tool assessment

To facilitate further benchmarking of newer tools, we have made all benchmark datasets available. This includes all input files, such as the genome fragments and the assembled simulated metagenomes. Files giving the taxonomic origins of all fragments and contigs are also available, to serve as an answer key when benchmarking new tools. The resulting classifications have been cleaned and compiled into a consistent format, such that classification results can be compared at the fragment/contig level without having to reclassify the input data. Additionally, all code used to analyze the data and generate figures is available for reference on GitHub,^2^ although modifications would have to be made to incorporate new tools, due to differences in output format, etc.

## 5. Conclusion and recommendations

**FIGURE 10:**
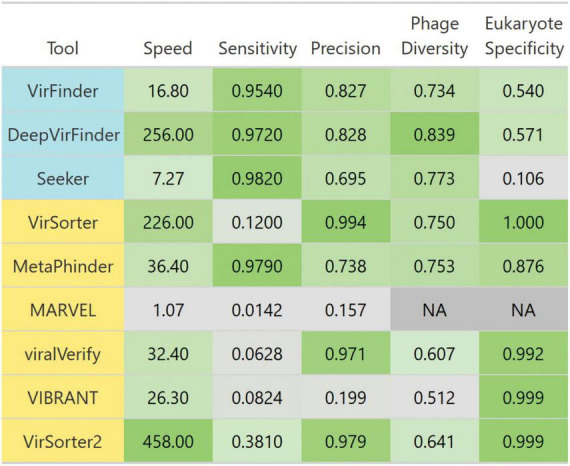
Breakdown of each evaluated tool’s performance. Tools are colored such that homology-based methods are yellow and sequence-based tools are blue. Colors are scaled from the minimum to maximum value in each column. Scales are linear, except for speed which is scaled to log 10 of speed. Speed is the average number of genome contigs from the *simulated metagenome set* classified per second. Sensitivity and precision are averages from classifying the simulated metagenomes set. Diverse phages is the average ratio of sensitivity on non-caudoviral phages vs. caudoviral phages. Eukaryote specificity is the average specificity when classifying eukaryotic genome fragments.

We summarized each tool’s performances for each benchmark challenge in Figure 10. Given these insights, the remaining question is “What is the best solution to phage detection and prediction?.” Unfortunately, answering this important question is not straightforward, especially given the tradeoffs between precision and sensitivity. However, some general guidelines can be used to decide which tools to use. For physically purified viromes (viral particle enriched metagenomes), precision is less of a concern, so one can prioritize sensitivity, and may choose DeepVirFinder, which also has the best sensitivity to non–caudoviral phages. For metagenomes where phages are actively infecting their bacterial hosts, the research question at hand should be the main driver in deciding. To identify novel phages, DeepVirFinder or Metaphinder may be a good choice, although the results should be further confirmed to avoid FPs, such as through the use of CheckV (Nayfach et al., 2021a), and when possible, host sequences should be removed prior to classification. However, if one is wanting to study the dominant phages present in an environment and maintain high confidence in phage calling, VirSorter2 or viralVerify may be good choices.

Given the high FPR for sequence-based tools, using several of these tools together to increase the sensitivity would lead to a decreased precision. Instead, a more robust multi-tool approach may combine sequence-based and homology based tools to find consensus predictions.

### 5.1. Limitations and future directions

This study aimed to set the basis for the development of a fair and reusable benchmark for viral detection tools. However, we wanted to highlight some limitations to the current study. First, we use the default parameters, reference databases, and trained models for all tools. While admittedly these may not be optimal, we believe that it is likely how a large proportion of researchers would use the tools. In particular, some tools allow users to choose a more stringent threshold to reduce false positives. The effect of such change in parameters can be investigated in this study by looking at the precision-recall curves provided in Supplementary material. However, the effect of further parameter tuning and database update was not evaluated in this benchmark and would be a valuable future effort.

Additionally, this benchmark did not investigate the tools’ ability to detect integrated prophage sequences nor plasmid sequences. Indeed, several tools such as VirSorter, VirSorter2. and VIBRANT are able to classify prophage sequences, and PPR-Meta is able to classify plasmid sequences. Future works may in particular evaluate the tool accuracy in detecting prophage boundaries and the effect of prophage inactivation and genetic degradation.

Second, all genomes used in the *genome fragment set* and simulated datasets were retrieved from RefSeq. This has a significant overlap with the tools’ reference databases and training sets (Table 1), therefore this study likely underrepresents the diminished performance on broader phage diversity.

Despite these limitations, we hope the developed benchmark may be informative to users and would be further developed to include new computational challenges. It should be noted that the results presented here are limited to those tools that could be installed and run by July 2021, and since then many more tools have been published [we are aware of 3CAC (Pu and Shamir, 2022), DeepMicrobeFinder (Hou et al., 2021), INHERIT (Bai et al., 2022), PHAMB (Johansen et al., 2022), PhaMer (Shang et al., 2022), VirMine 2.0 (Johnson and Putonti, 2022), and virSearcher (Liu Q. et al., 2022)]. Additionally, modular pipelines such as the IMG/VR viral discovery pipeline (Paez-Espino et al., 2017) and computational pipelines combining several tools presented here, were not evaluated in this work but could be assessed using the same benchmark datasets developed here. Importantly, the combination of several tools using different computational approaches could enable the researchers to leverage the strength of each approach. This type of hybrid pipeline has been in previously used by several large-scale viral discovery studies for human-associated metagenomes (Gregory et al., 2020; Camarillo-Guerrero et al., 2021; Nayfach et al., 2021b) and environmental metagenomes (Jian et al., 2021; Hegarty et al., 2022). With the potential that one of these tools or pipelines performs better than those studied, it would be useful to benchmark them using the data and methods developed here.

## Data availability statement

The datasets presented in this study can be found in online repositories. The names of the repository/repositories and accession number(s) can be found below: doi.org/10.5281/zenodo.7194616.

## Author contributions

KS: conceptualization, methodology, software, validation, formal analysis, investigation, data curation, writing—original draft, writing—review and editing, and visualization. JG: investigation, data curation, and writing—review and editing. AP: conceptualization, methodology, software, validation, investigation, resources, data curation, writing—review and editing, supervision, project administration, and funding acquisition. BH: conceptualization, resources, writing—review and editing, supervision, project administration, and funding acquisition. All authors contributed to the article and approved the submitted version.

## Footnotes

1
https://github.com/aponsero/Assembly_metagenomes

2
https://github.com/hurwitzlab/phage_detection_benchmarks
