# Supplementary material for: Evaluation of computational phage detection tools for metagenomic datasets
Source: Front Microbiol. 2023 Jan 25;14:1078760. doi: 10.3389/fmicb.2023.1078760 (PMC9902911; doi:10.3389/fmicb.2023.1078760)
Supplement: Supplementary file 1 [file Data_Sheet_1.pdf]

Supplementary Material

Supplementary Figures

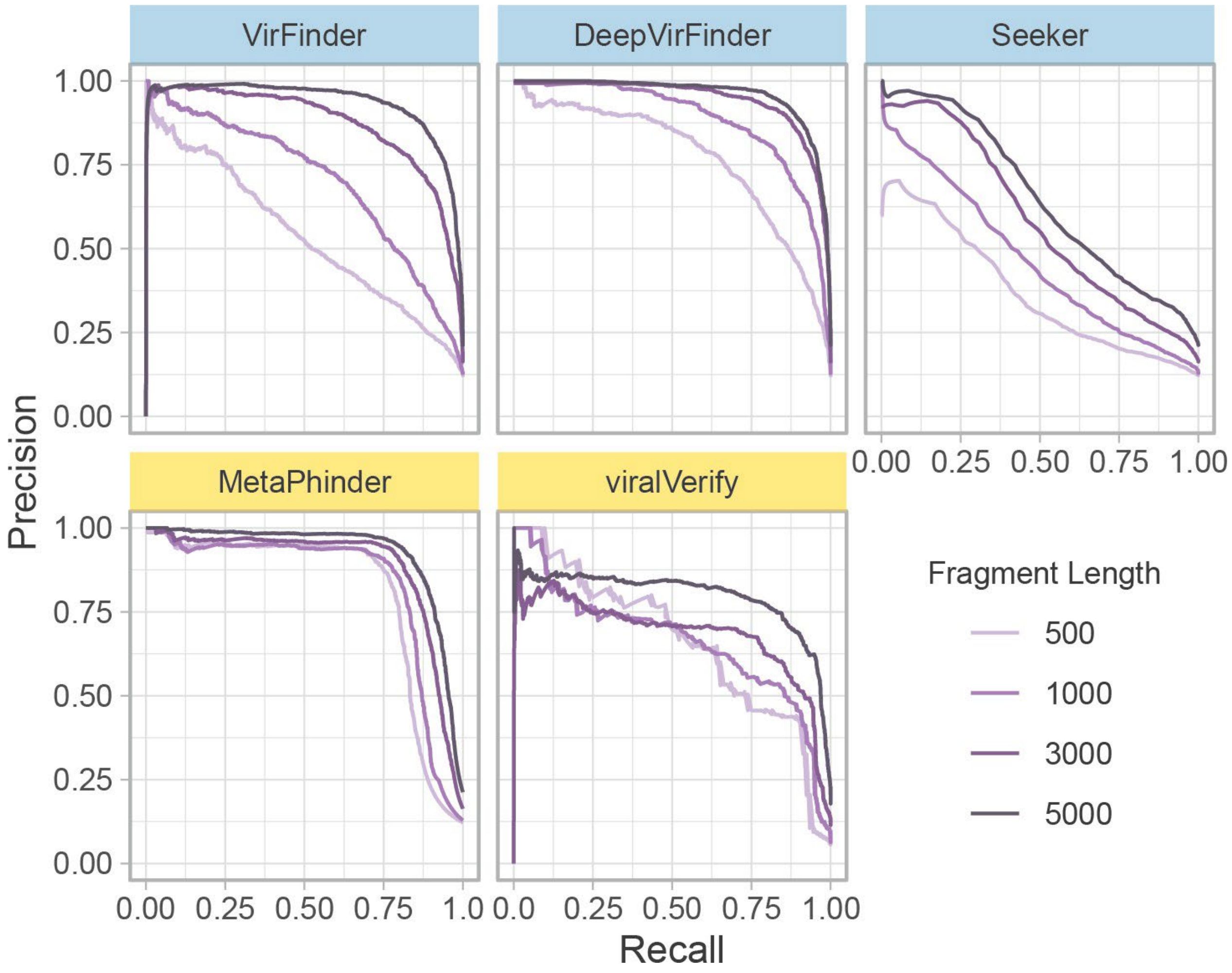

Supplementary Figure 1: Precision-Recall curves of tools for which a predicted value is available as an output. Only bacterial and phage fragments are included. Top row of tools are sequence-based (in blue), bottom two row is homology-based (in yellow).

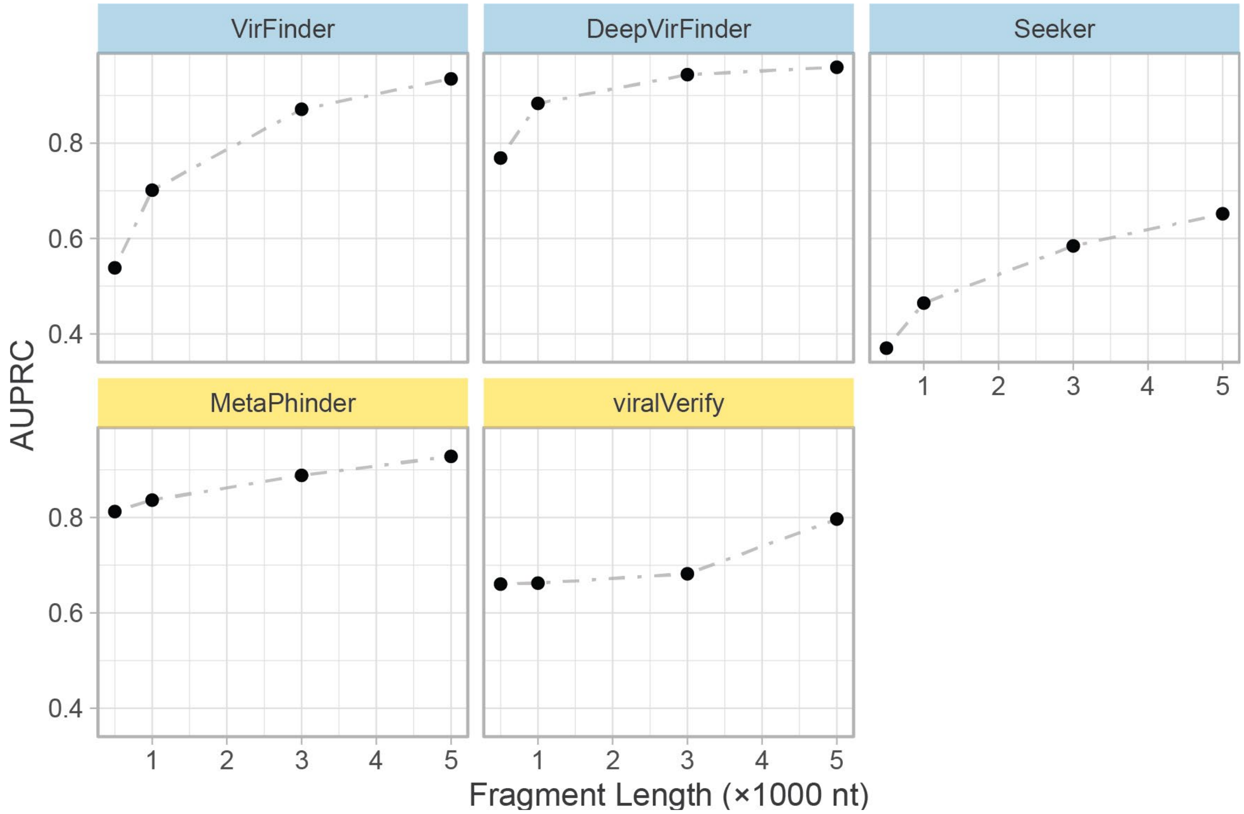

Supplementary Figure 2: Area under precision recall curve (AUPRC) vs fragment length of tools for which a predicted value is output. Only bacterial and phage fragments are included. Top row of tools are sequence-based (in blue), bottom row are homology-based (in yellow).

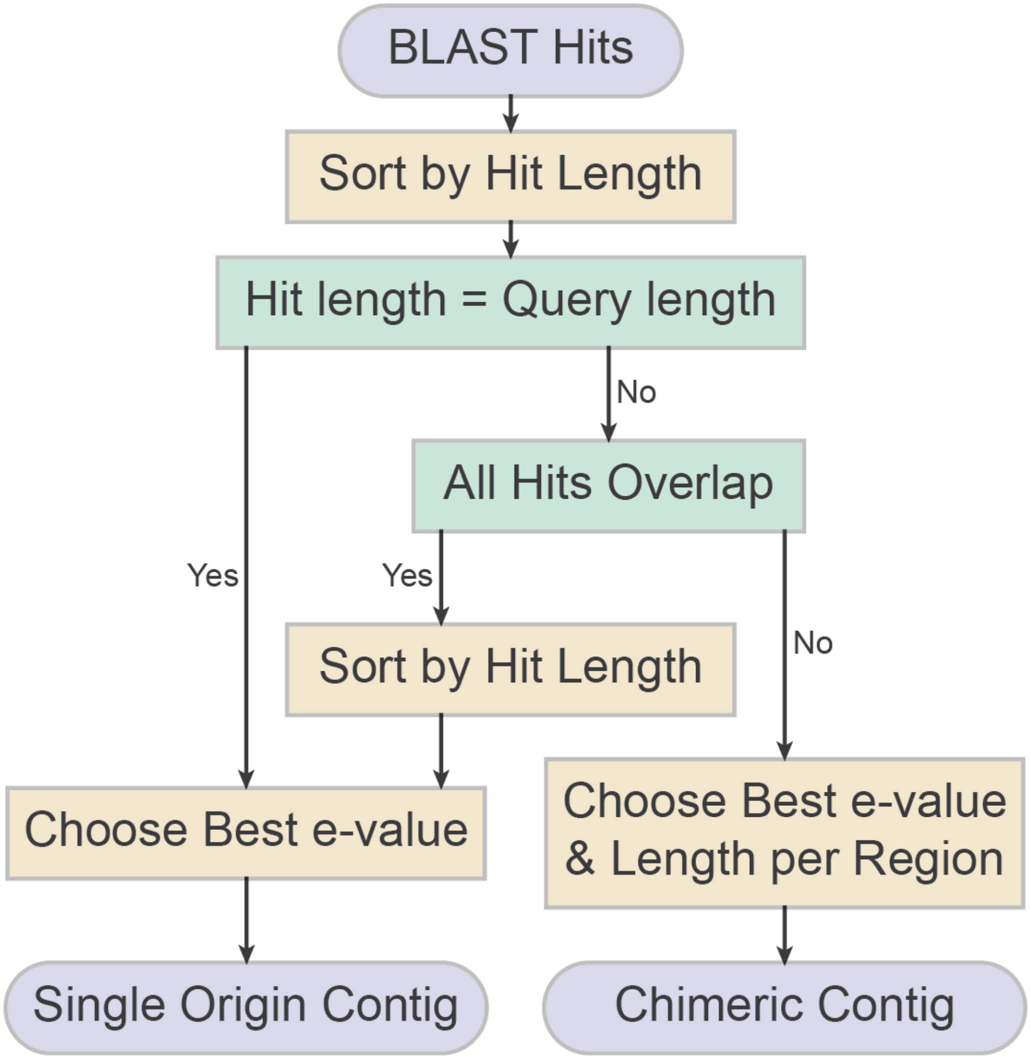

Supplementary Figure 3: Flowchart decision tree of how taxonomy of simulated contigs were assigned based on BLASTn search against a database of genomes used for read simulation.

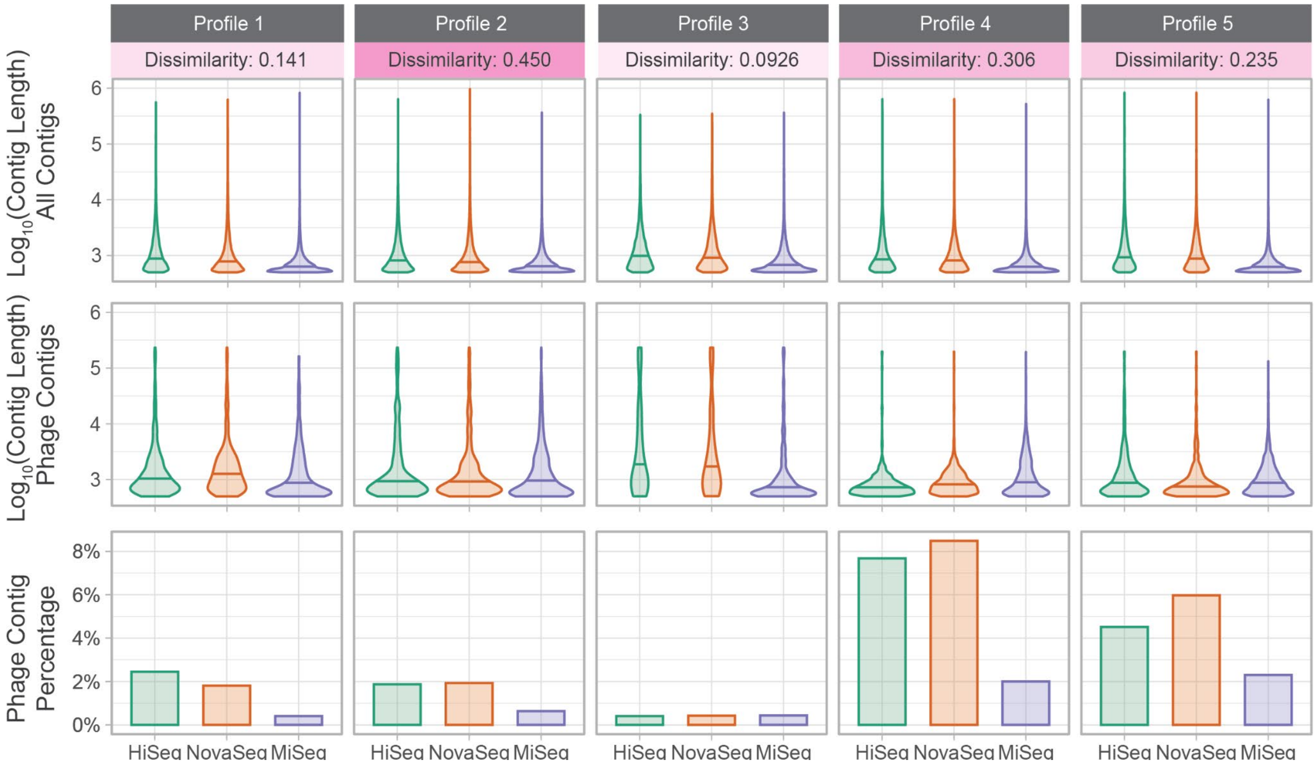

Supplementary Figure 4: Characteristics of simulated metagenomes generated by InSilicoSeq based on 5 marine abundance profiles using the 3 built-in error models (HiSeq, MiSeq, and NovaSeq). From top to bottom: Bray-Curtis dissimilarity between the original marine abundance profile and the abundance profile used for read simulation; Violin plots of (top row) all contigs, and (middle row) phage contig length distributions on log 10 scale with constant area violins and median marked with a horizontal line; Percentage of contigs that are from phages (bottom row).

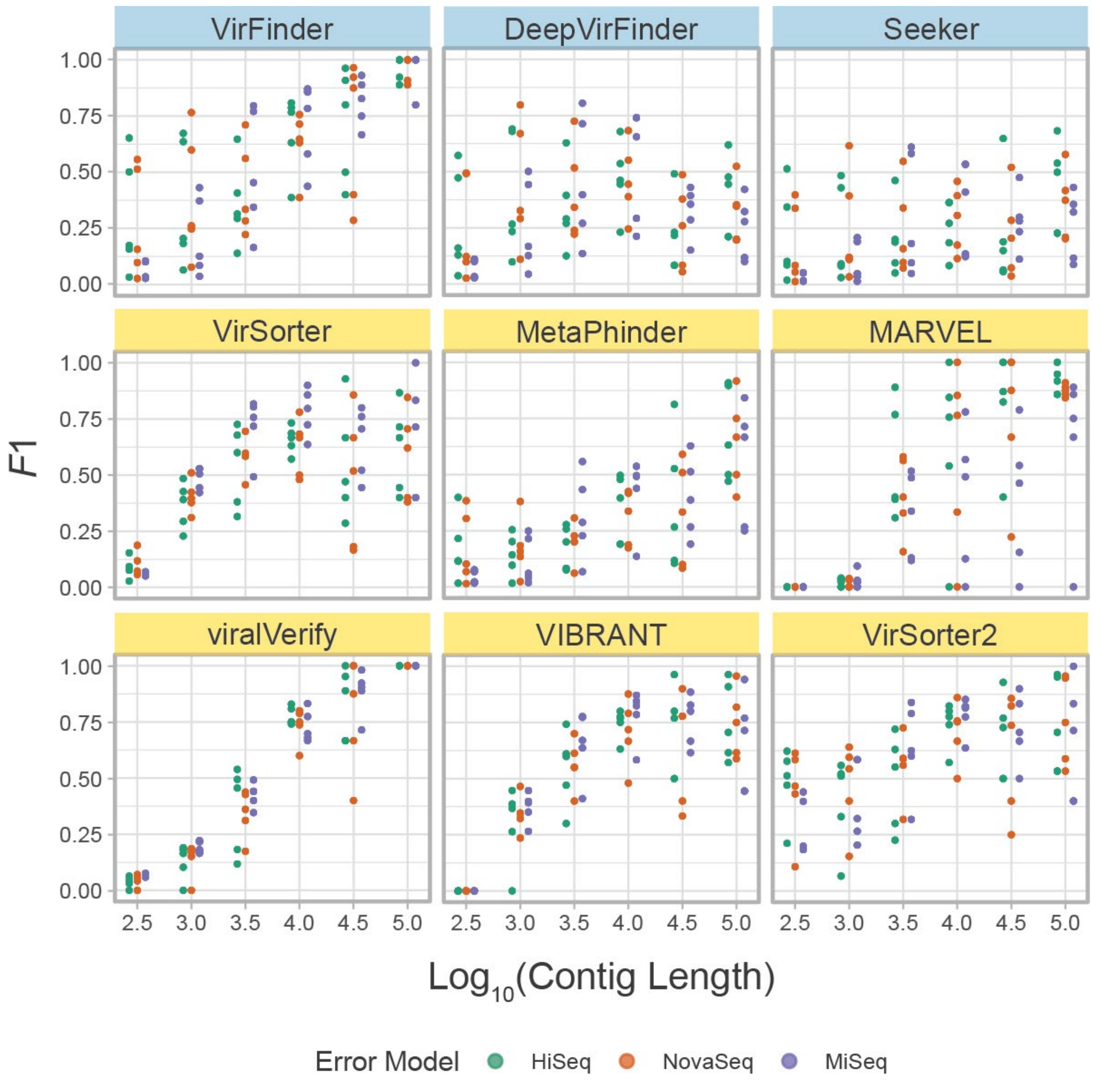

Supplementary Figure 5: *F*1 score for the classification of the simulated metagenome set vs log 10 of contig length. Top row of tools are sequence-based (in blue), bottom two rows are homology-based (in yellow).

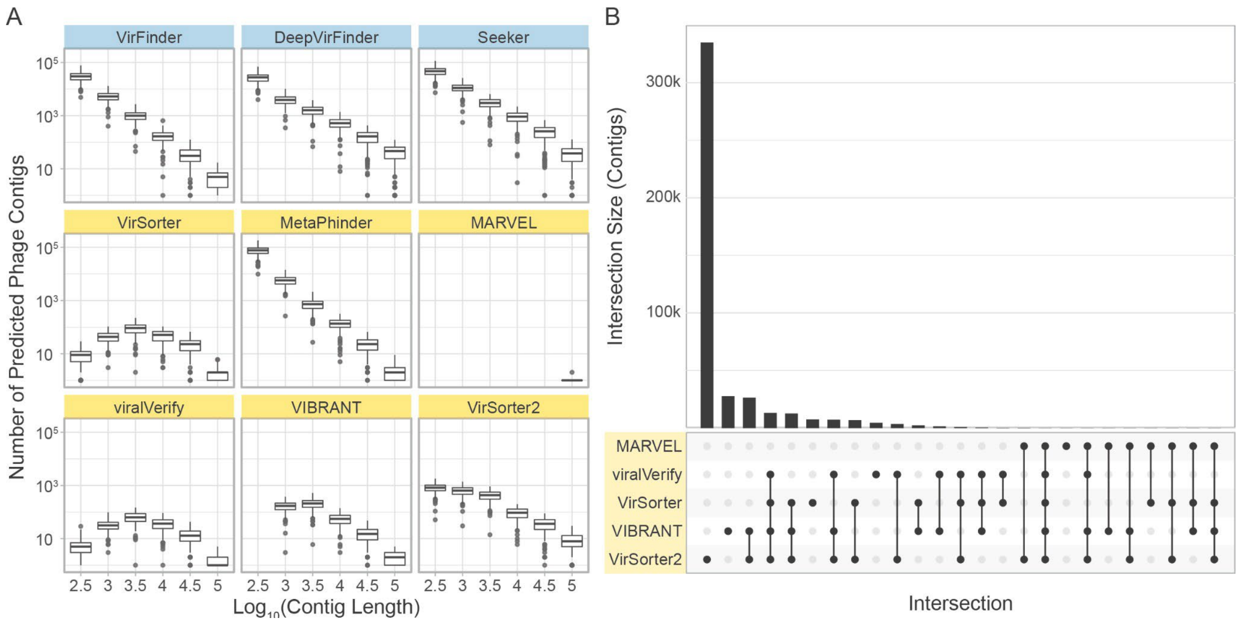

Supplementary Figure 6: Results of classifying *CRC dataset* A) Boxplot of the number of predicted phage contigs, on a log scale, in each length group per sample. B) Upset plot of homology-based tools, excluding MetaPhinder, showing intersection sizes in decreasing order. Homology-based tools are in yellow, sequence-based tools are in blue.

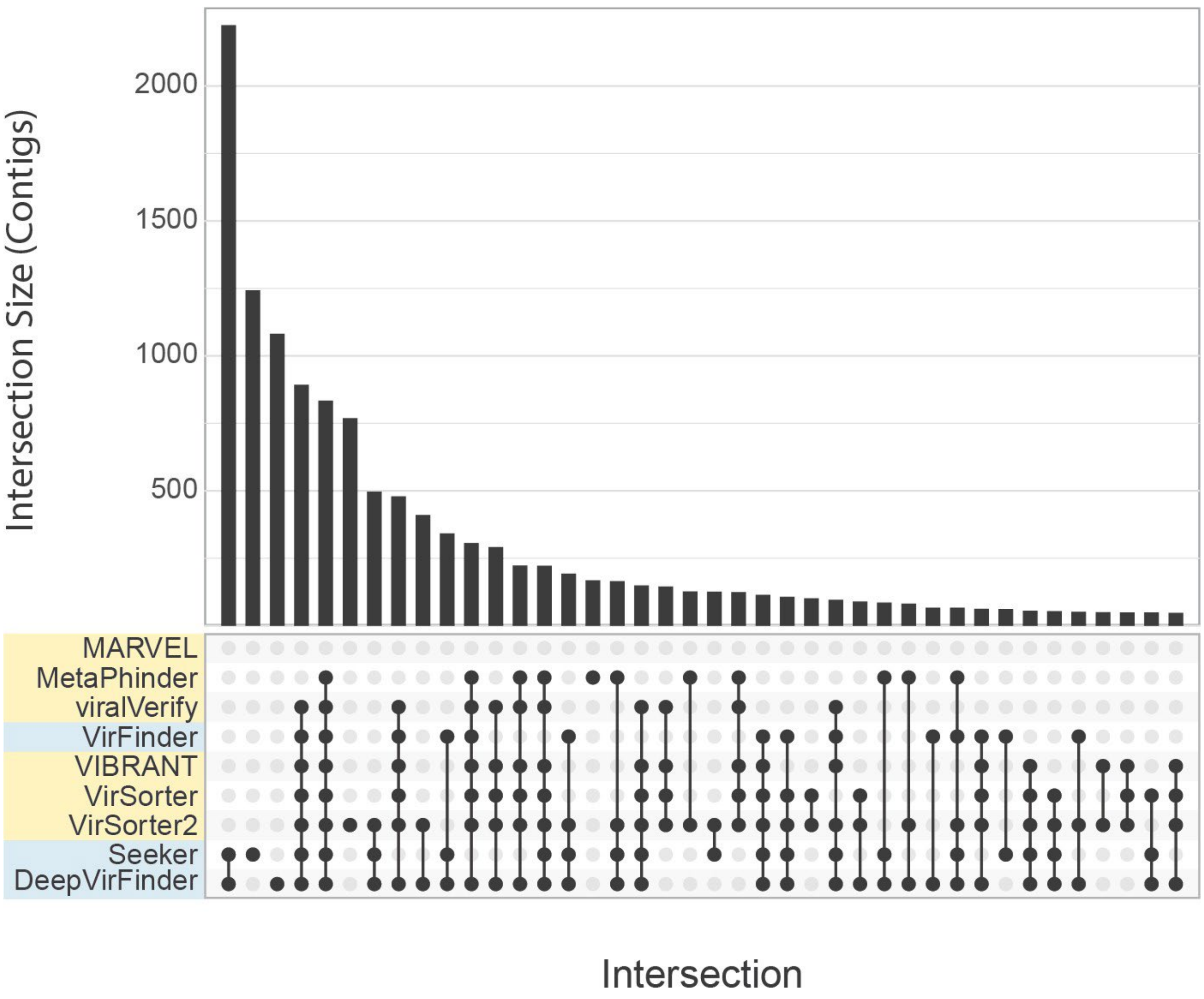

Supplementary Figure 7: Results from filtering predicted phage contigs from the CRC dataset, by including only those contigs labeled by CheckV as having Medium Quality, High Quality, or Complete virus. Upset plot showing the intersection sizes between the tools for contigs in one of those categories. Homology-based tools are in yellow, sequence-based tools are in blue.

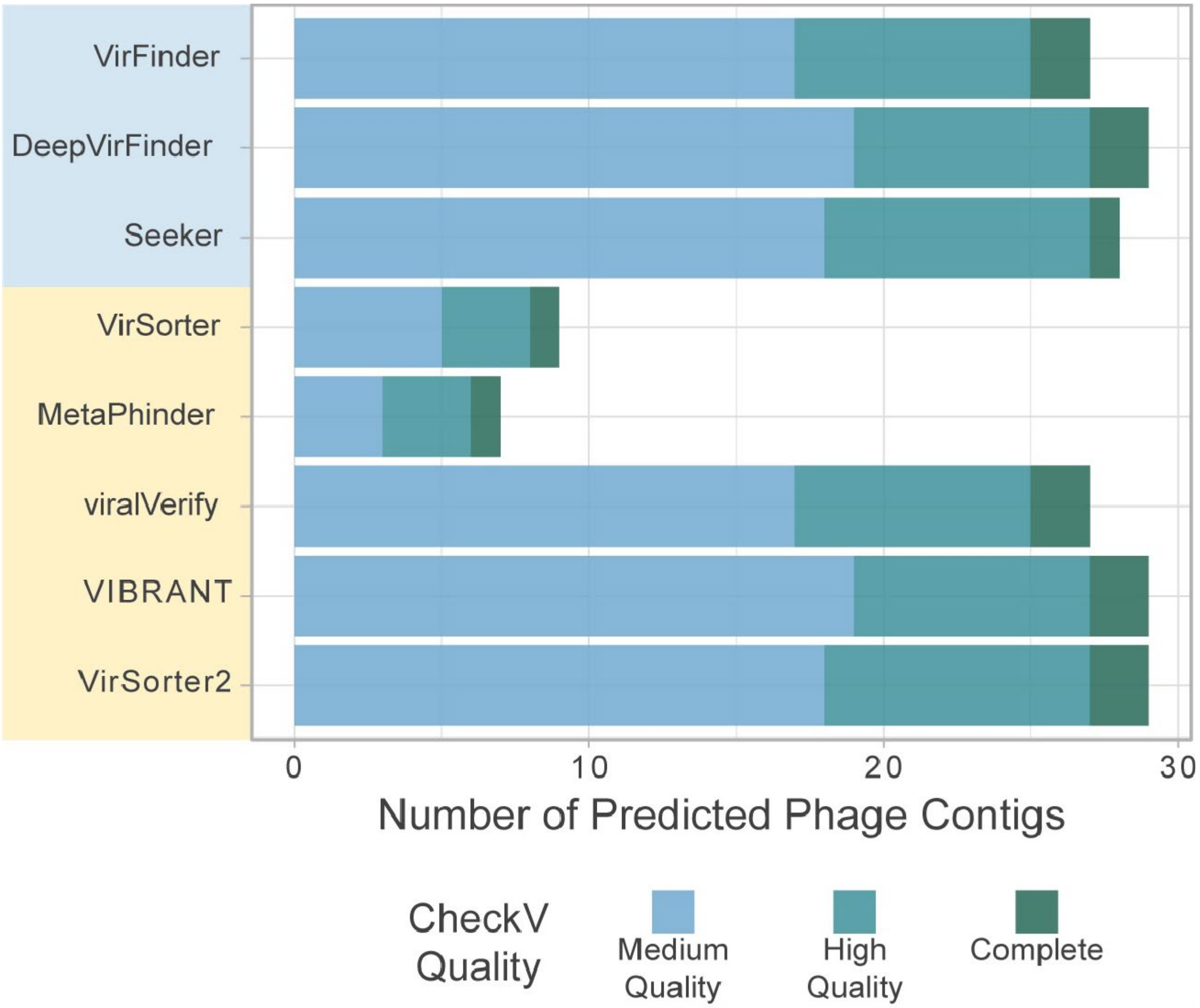

Supplementary Figure 8: Results of CheckV on the classified Gut Virome dataset. Predicted phage contigs of at least Medium quality were kept for this comparison.
